# Carbon Nanodots as Dual-Mode Nanosensors for Selective Detection of Hydrogen Peroxide

**DOI:** 10.1186/s11671-017-2214-6

**Published:** 2017-07-06

**Authors:** Cheng-Long Shen, Li-Xia Su, Jin-Hao Zang, Xin-Jian Li, Qing Lou, Chong-Xin Shan

**Affiliations:** 1 School of Physics and Engineering, Zhengzhou University, No. 75 Daxue Road, Zhengzhou, 450052 China; 2 State Key Laboratory of Luminescence and Applications, Changchun Institute of Optics, Fine Mechanics and Physics, Chinese Academy of Sciences, No. 3888 Dong Nanhu Road, Changchun, 130033 China

**Keywords:** Carbon nanodots, Fluorescence, Dual-mode nanosensors, Detection

## Abstract

Hydrogen peroxide (H_2_O_2_) is an important product of oxidase-based enzymatic reactions, such as glucose/glucose oxidase (GOD) reaction. Therefore, the probing of generated H_2_O_2_ for achieving the detection of various carbohydrates and their oxidases is very significative. Herein, we report one kind of dual-emission carbon nanodots (CDs) that can serve as novel dual-mode nanosensors with both fluorometric and colorimetric output for the selective detection of H_2_O_2_. The dual-model nanosensors are established only by the undecorated dual-emission CDs, where significant fluorometric and colorimetric changes are observed with the addition of different concentrations of H_2_O_2_ in the CD solution, which benefit to the achievement of the naked-eye detection for H_2_O_2_. The mechanism of the nanosensors can be attributed to the fact that the external chemical stimuli like hydroxyl radicals from H_2_O_2_ bring about the change of surface properties and the aggregation of CDs, which dominate the emission and absorption of CDs. The constructed dual-mode nanosensors exhibit good biocompatibility and high selectivity toward H_2_O_2_ with a linear detection range spanning from 0.05 to 0.5 M and allow the detection of H_2_O_2_ as low as 14 mM.

**Electronic supplementary material:**

The online version of this article (doi:10.1186/s11671-017-2214-6) contains supplementary material, which is available to authorized users.

## Background

Fluorescent carbon nanodots (CDs) have triggered extensive research attention for their unique physicochemical properties like good biocompatibility, low toxicity, tunable photoluminescence (PL), and high quantum yield. Because of the above characters, CDs have found potential applications in a variety of fields including but not limited to bioimaging, biosensors, and light-emitting devices [1–9]. Moreover, due to their up-conversion and down-conversion ability, lack of optical blinking, and high photostability compared to organic dyes or semiconductor quantum dots (QDs), CDs are more suitable for applications in fluorescent nanosensors by fluorescence increase or quenching [10–19].

Hydrogen peroxide (H_2_O_2_) is one kind of common oxidizer, which is always used as medical disinfectant for the ability of sterilization. Besides, H_2_O_2_ is also an important product of oxidase-based enzymatic reactions, such as glucose/glucose oxidase (GOD) reaction. Therefore, the sensing strategy through the probing of H_2_O_2_ can be employed as a promising approach for the detection of carbohydrates and their oxidases. For this reason, the sensing of H_2_O_2_ may be used to monitor the diseases about carbohydrate metabolism, such as diabetes. Currently, although various glucose sensors based on the determination of H_2_O_2_ have been developed by using a variety of analytical methods, previously reported sensor systems are mainly based on a single signal such as conductometric, fluorometric, or colorimetric change [20–22]. Recently, advances in nanotechnology, especially in fluorescent nanoparticles like semiconductor QDs and emerging carbon-based nanoparticles have brought about novel H_2_O_2_ nanosensors. Lu et al. developed one kind of dual-emission microhybrids (DEMBs) by combining CdTe QDs and rhodamine for ratiometric fluorescent sensing of glucose through monitoring the generation of H_2_O_2_ [20]. Zhang et al. reported a fluorescent nanosensor that showed selective and sensitive response to H_2_O_2_ through the fluorescence quenching of CDs [21, 22]. However, these work inevitably brought about the intrinsic defects of semiconductor-based QDs with expensive chemical constituents and heavy metal pollution. Moreover, the nanosensors based on single signal readout, either fluorescence quenching or color change, may have poor assay stability due to the fluctuations of environmental factors and the experimental operation errors. On account of the above consideration, we wish to develop a new class of fluorescent CDs, whose fluorescence and solution color are very sensitive to the change of the concentrations of H_2_O_2_. Thus, a dual-mode nanosensor based on these CDs can be achieved for distinctively and sensitively sensing the H_2_O_2_ by simultaneously inspecting the fluorometric and colorimetric changes of CD solution, which is beneficial to the realization of naked-eye detection of the H_2_O_2_.

In this study, we have developed a facile and convenient method to synthesize a novel type of CDs, which exhibits a dark red solution color under visible light and dual fluorescent emission under a 365-nm UV lamp (blue and green fluorescence emission). The CDs are simply synthesized through solvothermal method with citric acid, urea, and *N*,*N*-dimethylformamide (DMF) as carbon source, nitrogen source, and reaction solvent, respectively. The fluorescence and solution color are very sensitive to changes in the concentrations of H_2_O_2_. Thus, a dual-mode nanosensor based on these CDs can be achieved for distinctively and sensitively sensing the H_2_O_2_ by simultaneously inspecting the fluorometric and colorimetric changes of the CD solution, which is beneficial to the realization of naked-eye detection of the H_2_O_2_. Without the introduction of any expensive instrument, a dual-mode nanosensor based on these CDs has been established. This sensing system may effectively avoid the potential operation errors and markedly improve the reliability of the measurement. In addition, the CD-based nanosensors are promising in the application of blood glucose detection both in vivo and in vitro owing to their good biocompatibility and high water solubility.

## Methods

### Synthesis of CDs

The CDs were prepared using a solvothermal method with citric acid as the carbon source, urea as the nitrogen source, and DMF as the co-reactant. In a typical experiment, citric acid (1 g) and urea (2 g) were dissolved in 10 mL DMF. The solution was then transferred to a 25-mL poly(tetrafluoroethylene)-lined autoclave and heated at 160 °C for 4 h. After the reaction, the autoclave was naturally cooled down to room temperature. A dark red solution was obtained. The CDs were precipitated by adding 5 mL reaction solution into 25 mL abundant ethanol and centrifuged at 7500 rpm for 30 min. Then, the precipitation was dialyzed to obtain pure CDs. The as-prepared CDs were collected and dried in a vacuum drying oven at 60 °C and under <1 Pa for 12 h. Then, the CDs were redissolved in deionized water to form 0.75 mg mL^−1^ CD solution for further research. And the subsequent H_2_O_2_-treated CDs were collected and dried with the same method for the characterization of the surface morphology and structural properties.

### Measurements

The surface morphology of the CDs was characterized by a high-resolution transmission electron microscope (HRTEM, JEOL JSM-IT100). The structural properties of the CDs were performed by an X-ray diffractometer (XRD, PA National X’Pert Pro) and a micro-Raman spectrometer (Renishaw RM 2000). The absorption spectra of the CDs were measured on a Hitachi U-3900 UV-Vis-NIR spectrophotometer. The fluorescence spectra of the CDs were measured by a spectrophotometer (Hitachi F-7000). The fluorescence quantum yield of the CDs was obtained by the Horiba FL-322 spectrometer with a calibrated integrating sphere. The fluorescence decay curves of the CDs were also measured by Horiba FL-322 using a 405-nm NanoLED monitoring the emission at 450 and 500 nm, respectively. The Fourier transform infrared spectrum (FTIR) of the CDs was recorded on a Bio-Rad Excalibur spectrometer (Bruker Vector 22). X-ray photoelectron spectroscopy (XPS) was recorded on an ESCALAB MK II X-ray photoelectron spectrometer using Mg as the exciting source.

### Establishment of the CD Nanosensors

For the detection of the H_2_O_2_, the fluorescence and absorption spectra of the CDs in the presence of H_2_O_2_ were examined in PBS buffer (pH = 7.4, at 25 °C). In a typical experiment, a different amount of H_2_O_2_ was mixed with distilled water firstly and then 20 μL 0.75 mg mL^−1^ CD solution was injected into 4 mL H_2_O_2_ solution with different concentrations (0, 0.05, 0.1, 0.15, 0.25, 0.5, 1.0, and 2.0 M). Then, photographs, fluorescence, and absorption spectra were taken after the CDs were added into the H_2_O_2_ solution.

The selectivity of the CD-based nanosensors was also evaluated. The CD solution (20 μL, 3.75 μg mL^−1^) was mixed with different kinds of cations and oxidants (4 mL, 0.1 M) and then the solution was shaken for 1 min. At last, the UV-Vis absorption and fluorescence spectra of the solution were recorded after the CDs were added into the H_2_O_2_ solution.

## Results and Discussion

### Characterization of the CDs

**Fig. 1:**
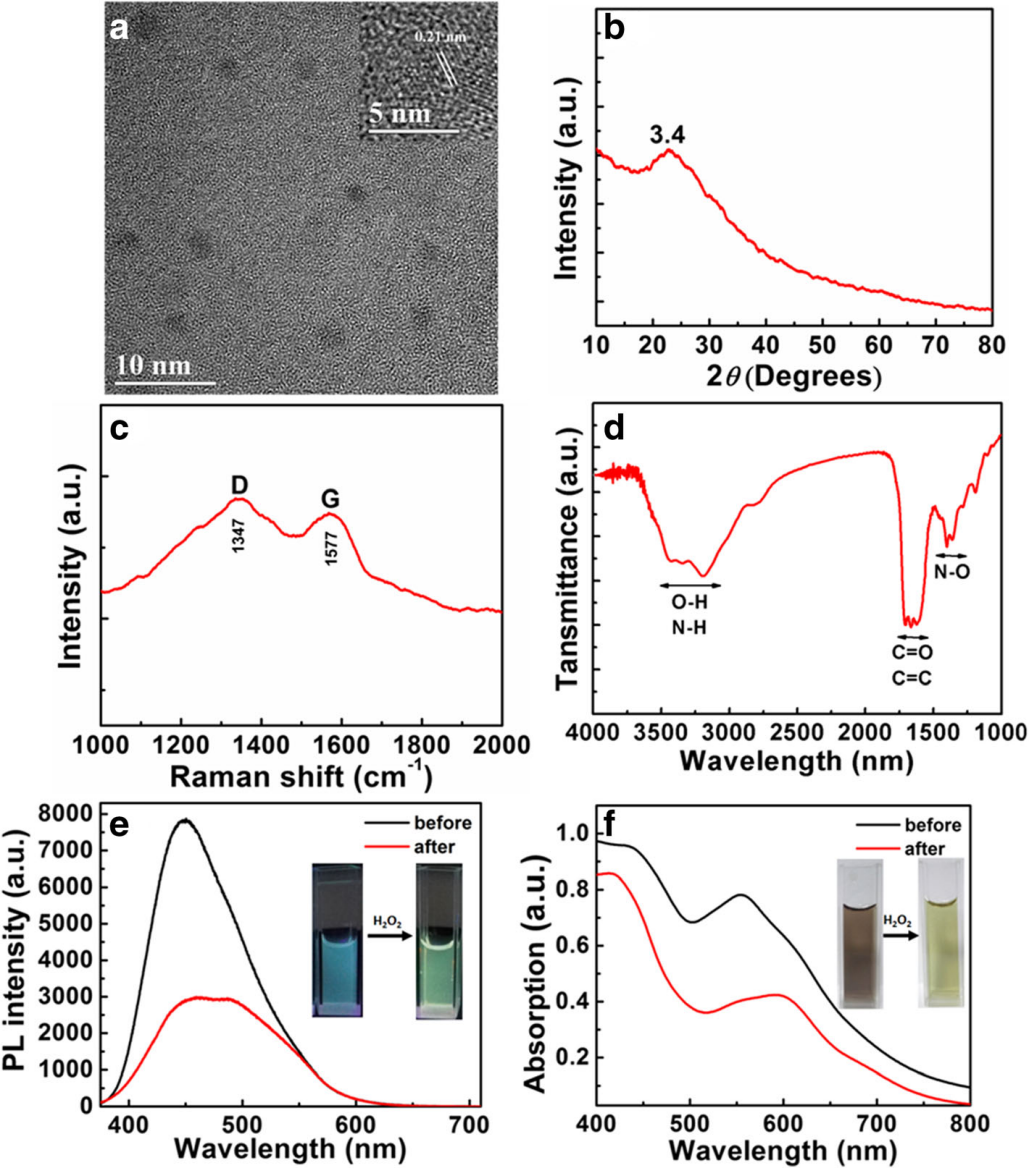
**a** TEM image of the CDs. *Insets* show the HRTEM image of the CDs. **b** XRD pattern of the CDs. **c** Raman spectroscopy of the CDs. **d** FTIR spectroscopy of the CDs. **e** Fluorescence variation of CDs after adding 0.5 M H_2_O_2_. *Insets* show photographs of CDs before (*left*) and after (*right*) adding the H_2_O_2_ under UV light. **f** Colorimetric variation of CDs after adding 0.5 M H_2_O_2_. *Insets* show photographs of CDs before (*left*) and after (*right*) adding the H_2_O_2_ under daylight

The morphology of the as-prepared CDs was measured by transmission electron microscope (TEM). As shown in Fig. 1a, the CDs are well dispersed with a uniform size range of 2.5–6.5 nm and an average diameter of around 5 nm (Additional file 1: Figure S1b). Moreover, the HRTEM image (inset of the Fig. 1a) shows the diffraction fringes around 0.21 nm which agrees with the (100) of graphite. The XRD patterns of the CDs shown in Fig. 1b exhibit a broad peak at around 23.4°, which is corresponding to the highly disordered carbon atoms with a graphite-like carbon structure. The Raman spectra of the CDs (Fig. 1c) reveal two bands: D band (at around 1347 cm^−1^, which was due to the vibrations of sp^3^-hybridized carbon with imperfection and disorder) and G band (at around 1577 cm^−1^, which was associated with the E_2g_ vibration modes of sp^2^-hybridized carbon in a two-dimensional hexagonal crystalline structure). The FTIR spectra of the CDs (Fig. 1d) present broad vibration absorption bands of O–H/N–H at 3100–3600 cm^−1^, the stretching vibrations of C=O/C=C at around 1690–1610 cm^−1^ and the stretching vibrations of N–O at around 1350–1390 cm^−1^. The above data indicate that there may be some functional groups on the surface of the CDs, and these functional groups may play an important role in the high hydrophilicity and stability of the CDs in aqueous solution.

The fluorescent behavior of the CD-based nanosensors toward H_2_O_2_ was measured in the CD aqueous solutions shown in Fig. 1e. Under a single wavelength excitation at 365 nm, the CD solutions illustrate asymmetrical emission spectra, which could be fitted by dual-emission fluorescent bands centered at 450 and 500 nm, corresponding to blue and green fluorescent bands, respectively. When the CD solutions are mixed with H_2_O_2_, the intensity of the blue band demonstrates a greater decrease than that of the green one. Accordingly, the strongest emissions of the CDs shift from 450 to 500 nm from the results of the excitation-emission matrices of the CDs after the addition of the H_2_O_2_ (Additional file 1: Figure S2). As a result, the fluorescence color of the CD solutions changes from blue to green under a 365-nm UV lamp illumination (inset of the Fig. 1e). Moreover, the CD solutions simultaneously experience a colorimetric change from dark red to green after adding the H_2_O_2_ (inset of the Fig. 1f). This color change can be attributed to the intensity evolution of the absorption bands at around 555 and 595 nm caused by the addition of H_2_O_2_ in the CD solution (Fig. 1f). Taken together, these results confirm the CDs could be used as a dual-mode nanosensor for the detection of H_2_O_2_.

### Sensing Mechanism

To investigate the sensing mechanism, the morphology and fluorescence properties of the CDs after adding H_2_O_2_ were also characterized. As illustrated in Additional file 1: Figures S1a and S1c, the addition of H_2_O_2_ into the CD solution can lead to the aggregation of CDs, whose sizes are ranging from 30 to 60 nm. The H_2_O_2_-induced aggregation of CDs was also revealed in the normalized absorption spectra (Additional file 1: Figure S3); namely, the absorption band of the CDs red-shifts from 555 to 595 nm in the visible region [15]. Correspondingly, the color of the CD solution varies from dark red to green, along with the dispersion state of CDs turning into an aggregation state. The XRD spectra (Fig. 1b and Additional file 1: Figure S4) of the CDs before and after adding H_2_O_2_ alter little, indicating there are no changes in the crystalline structure of the CDs.

**Fig. 2:**
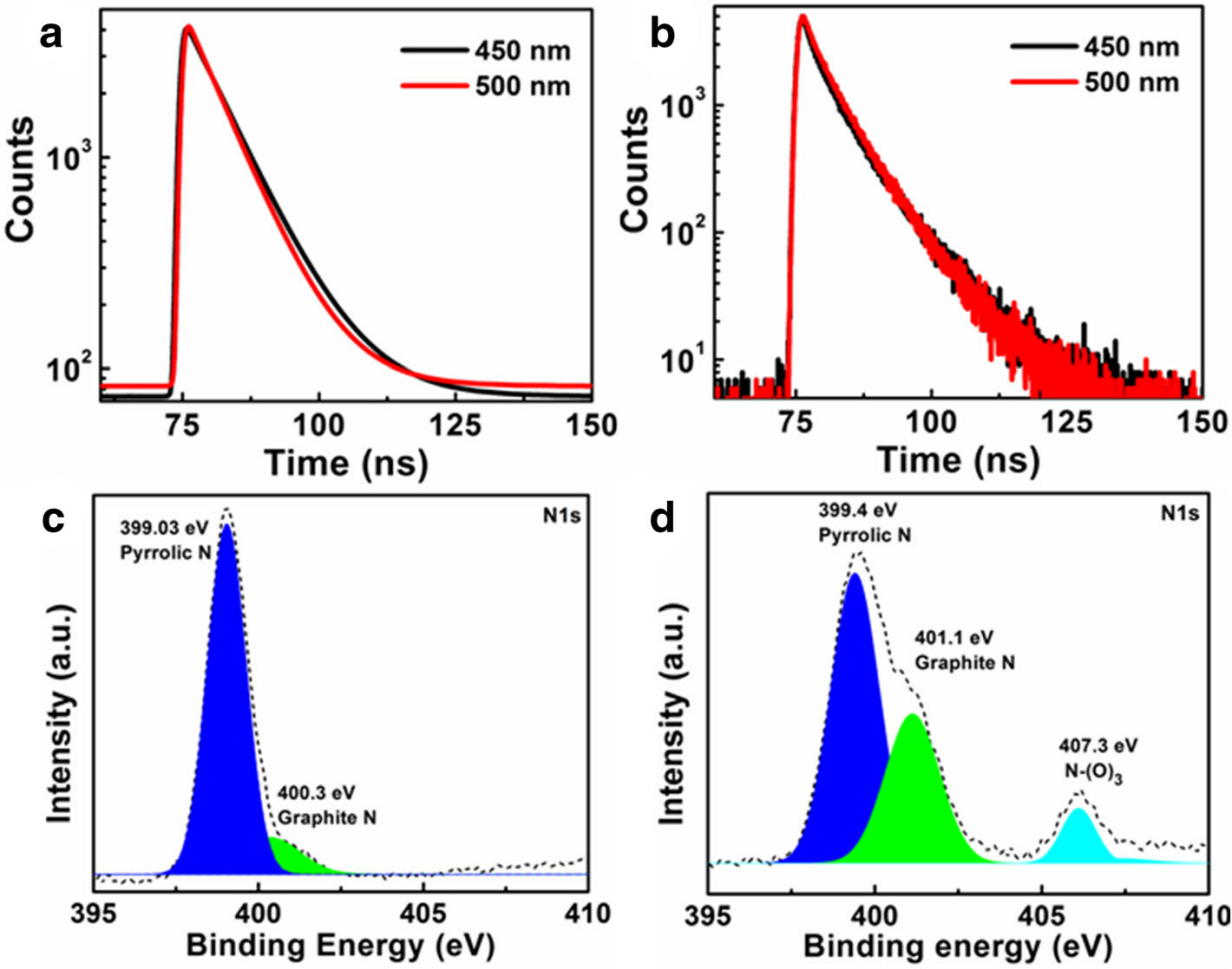
**a**, **b** Fluorescence decay of CDs before (**a**) and after (**b**) adding 0.5 M H_2_O_2_. **c**, **d** XPS (N1s) of CDs before (**c**) and after (**d**) adding 0.5 M H_2_O_2_

The fluorescence evolution of the as-prepared CDs with the addition of H_2_O_2_ was investigated by fluorescence spectra. The excitation-emission matrices show that the addition of H_2_O_2_ makes the emission centers of the CDs change from 450 to 500 nm (Additional file 1: Figure S2). The fluorescence decay curves shown in Fig. 2a for the CDs with the emission at 450 and 500 nm can be well fitted by a mono-exponential decay function with an average lifetime of 7.96 and 7.12 ns, respectively (under excitation of 365 nm). In contrast, the fluorescence decay lifetime of the CDs after the H_2_O_2_ treatment turned into 4.53 and 4.83 ns (Fig. 2b and Table 1). Meanwhile, the PL quantum yield (*η*_int_) of CDs changed from 5.5 to 4.6% when the H_2_O_2_ was added in the CD solution. Considering the change of fluorescence lifetime and PL quantum yield, it can be concluded that charge transfer (CT) may occur between CDs and H_2_O_2_, which could be a trigger to make the change of PL spectra of the CDs.

**Table 1:** Photophysical data for CDs (3.75 μg mL^−1^ in deionized water) before and after 0.5 M H_2_O_2_ treatment

States	*λ*_em_^a^ (nm)	*τ* ^b^ (ns)	*χ* ^2c^	QY^d^ (%)
Before	450	7.96	0.999	5.5
Before	500	7.12	0.999	
After	450	4.53	0.993	4.6
After	500	4.83	0.996	

^a^PL peak excited at 405 nm

^b^PL lifetime

^c^Goodness of fit

^d^PL quantum yield excited at 365 nm

The FTIR and XPS spectra of the CDs were measured to give insight into the chemical composition and environmental changes caused by the H_2_O_2_. The FTIR spectra of CDs before and after adding H_2_O_2_ shown in Additional file 1: Figure S7 illustrate that the stretching vibrations of N–O at around 1350–1390 cm^−1^ increase with the addition of H_2_O_2_, which is also confirmed by the result of the XPS spectra. It is observed from the full survey XPS spectra (Additional file 1: Figure S8) that the O to N ratio of the CDs before and after the H_2_O_2_ treatment was 1.57 and 3.85, respectively. The increasing ratio of O/N reveals that the bonding states of N in the CDs may change with the addition of the H_2_O_2_, which is in line with the high-resolution N1s XPS spectra shown in Fig. 2c, d. From the result of the N1s XPS spectra, the content of graphite N in the CDs has been increased with the addition of the H_2_O_2_. Furthermore, there is an additional peak of N–O state at 407.3 eV in the N1s spectra after the addition of the H_2_O_2_, which obviously demonstrates that the importing of the H_2_O_2_ brings about the variation of the surface states in the CDs. All the surveys manifest that the surface N frame could be changed by the addition of the H_2_O_2._

**Fig. 3:**
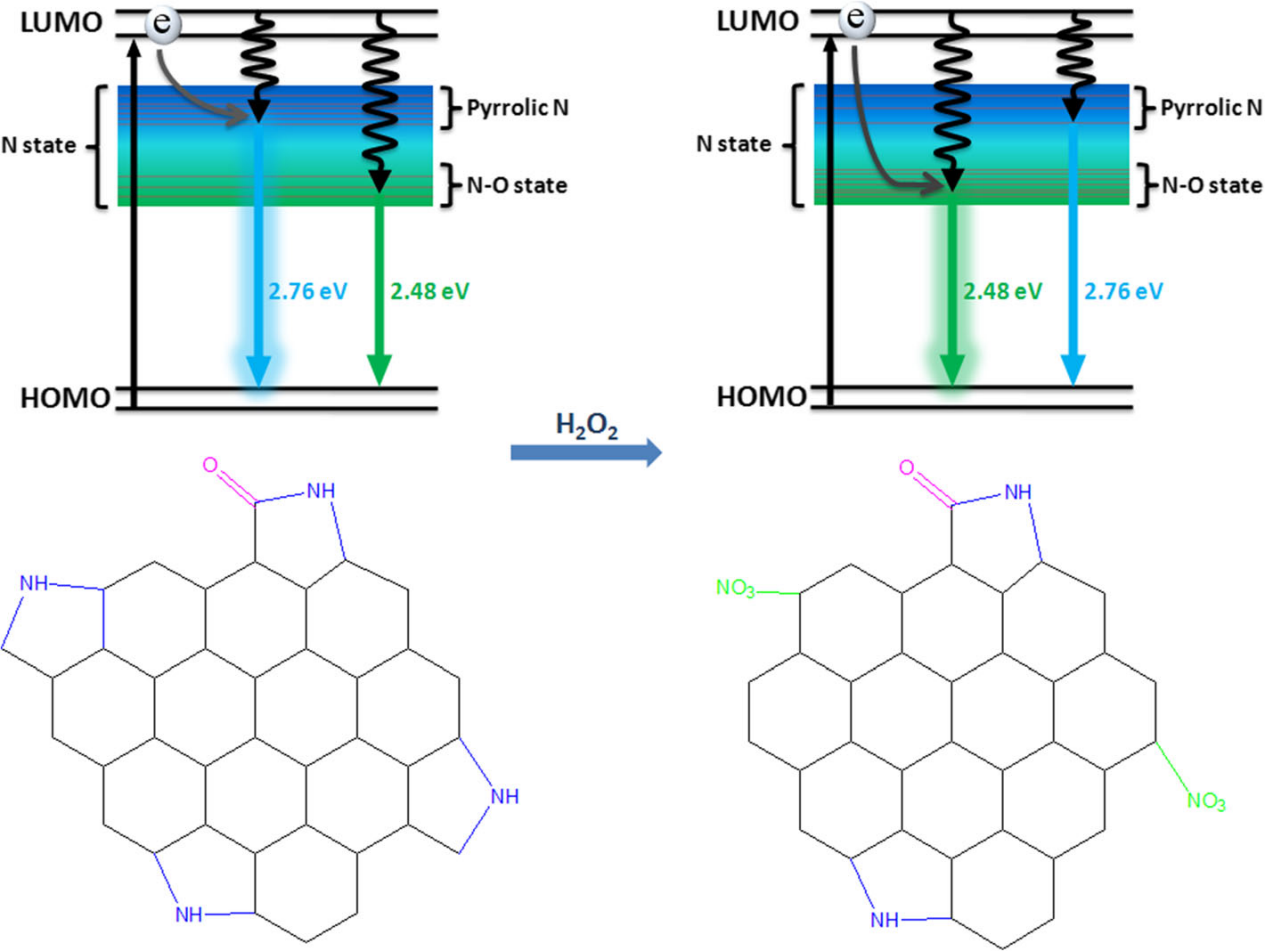
The possible sensing mechanism for CDs before (*left*) and after (*right*) adding H_2_O_2_

Previous reports suggest that the emission bands of the CDs are related to the surface states such as N-doped radicals and urea groups [5, 9, 12, 23–25]. Meanwhile, these surface states are sensitive to external physical or chemical stimuli. On the basis of the photophysical and surface environmental analysis, we propose the mechanism of the fluorescence evolution with the introduction of the H_2_O_2_ (Fig. 3). The edge state of the as-prepared CDs is consisted of the conjugated pyrrolic N groups. This type of N state may be mostly localized at high energy level. Thus, the excited electron may nonradiatively relax to the high-level surface N state and then radiatively transfer to ground state accompanied with fluorescence emission bands around 450 nm. In contrast, the fluorescence intensity of the CD solution slightly decreases because of the dynamic quenching between the H_2_O_2_ and the CDs, where the CT arises between CDs and H_2_O_2_ similar to the previous reports [26–29]. Otherwise, it could be deduced that the high-energy fluorescence radicals (related N state) are transformed into the lower-energy N–O state in virtue of the impact of hydroxyl radical from the H_2_O_2_. So, the excited electron may mostly relax with a radiative transition from the lower-energy N–O state to ground state with a green emission band at 500 nm, which also results in the static quenching of 450-nm fluorescence. Therefore, the major emission bands of CDs could present a change from the blue emission to the green emission.

### Evaluation of the CDs Nanosensors

On the basis of the above fluorescent and colorimetric behavior of the CDs, we have developed a nanosensor to detect H_2_O_2_ by the CDs. The proposed sensing system is consisted of CDs with proper concentration in aqueous solution (3.75 μg mL^−1^, Additional file 1: Figure S9), where the CDs serve dual function as both colorimetric and fluorometric reporters in this system.

**Fig. 4:**
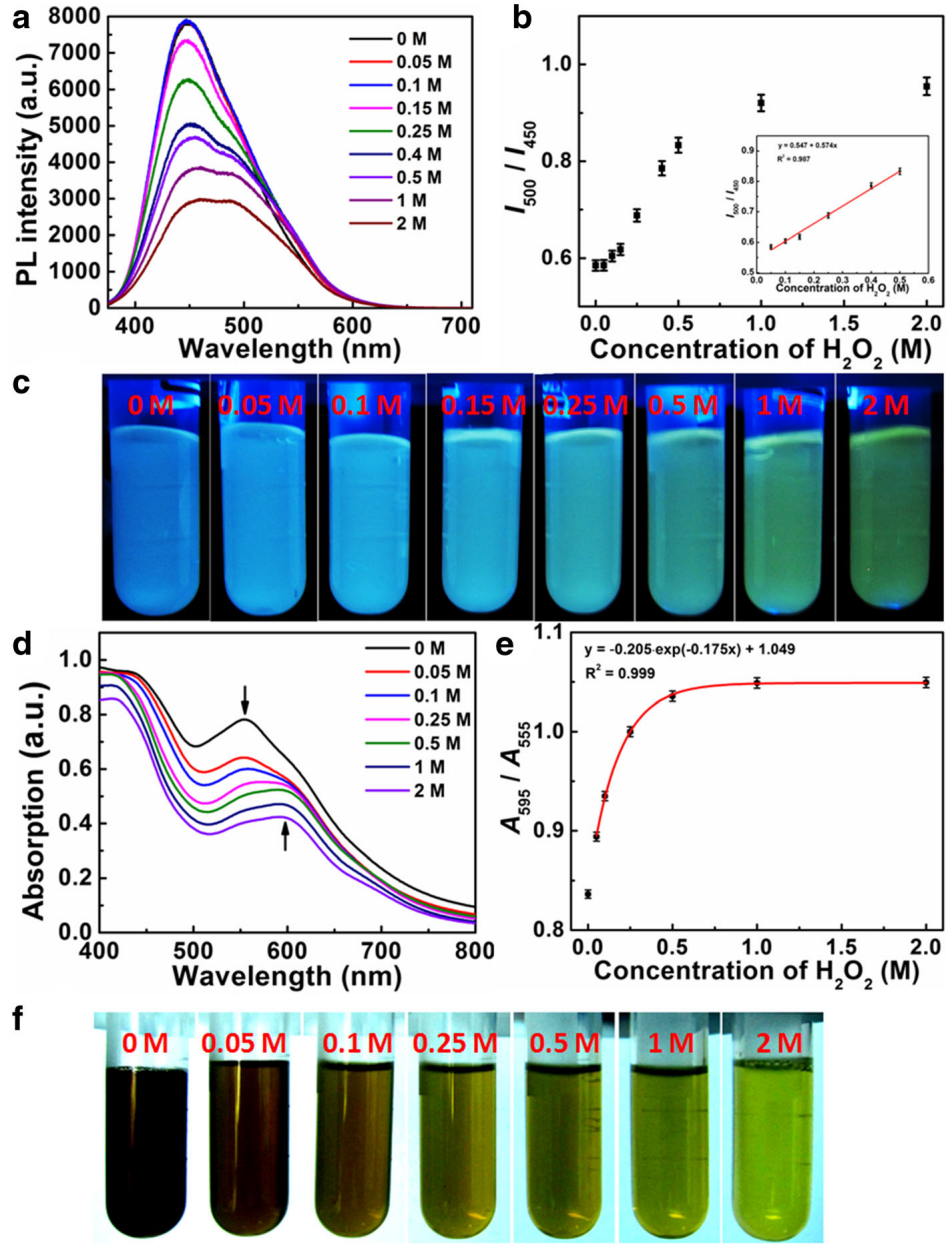
**a** Fluorescence spectra of CDs in the presence of different H_2_O_2_ concentrations. **b** Calibration curve of *I*_500_/*I*_450_ of the CDs vs. H_2_O_2_ concentration. *Insets* show the linear detection range of *I*_500_/*I*_450_ of the CDs vs. H_2_O_2_ concentration. **c** Photographic images of the fluorescence CD solution under different concentrations of H_2_O_2_. **d** UV-Vis spectra of CDs in the presence of different H_2_O_2_ concentrations. **e** Calibration curve of *A*_595_/*A*_555_ of the CDs vs. H_2_O_2_ concentration. **f** Photographic images of the CD solution under different concentrations of H_2_O_2_

The proposed nanosensing system based on the CD solution is illustrated in Fig. 4. The fluorometric and colorimetric change caused by H_2_O_2_ could be distinctly visualized by the naked eye (Fig. 4c, f), where a series of noticeable color change from blue to green and from dark red to green can be observed under UV light and daylight illumination. Besides, the addition of H_2_O_2_ into the CD solution can also be expressed quantitatively with the fluorescence and absorption spectra (Fig. 4a, d). As shown in Fig. 4a, the fluorescence band centered at 450 and 500 nm decreases gradually with the increase of the H_2_O_2_ concentration from 0 to 2 M. However, the increase of the H_2_O_2_ concentration leads to the different decrease of fluorescence intensity at 450 nm (*I*_450_) and 500 nm (*I*_500_), which accords well with the fluorescence color change in the CD solution (Fig. 4c). Therefore, the ratio of the fluorescence intensity at 500 nm to that at 450 nm is selected to monitor the H_2_O_2_ concentration (Fig. 4a, b). The lower ratio is related to the blue emission, while the green fluorescence can be observed by the naked eyes at a higher ratio of *I*_500_ to *I*_450_. The linear detection range by these means spans from 0.05 to 0.5 M with a linear correlation *R* ^2^ = 0.987. Similarly, the colorimetric change occurs in the CD solution on account of the inhomogeneous decrease of the absorption band at 555 and 595 nm. As displayed in Fig. 4d, the absorption intensity decreases in the visible region, but the increase of the H_2_O_2_ concentration results in that the absorption around 595 nm decreases more slowly than around 555 nm. Hence, the ratio of the absorption at 595 nm (*A*_595_) to that at 555 nm (*A*_555_) could be also used to measure the H_2_O_2_ concentration. The ratio of *A*_595_ to *A*_555_ increases exponentially from 0.05 to 2 M with an exponential correlation *R* ^2^ = 0.999, and the colorimetric change correlates well to the H_2_O_2_ concentration range from 0.05 to 0.25 M with the linear detection limit (LOD) of 14 mM (Additional file 1: Figure S11 and Table S1). The dual-mode nanosensors have a proper sensitivity of this method satisfying the clinical and medicinal requirements due to the concentration of H_2_O_2_ through the GOD reaction ranging around millimolar (~10 mM) in plasma [20]. In addition, the dual-mode nanosensors have an intrinsic built-in calibration reference, so intensity fluctuation and other externally caused factors can be eliminated, which contributes to the promotion of the testing accuracy.

**Fig. 5:**
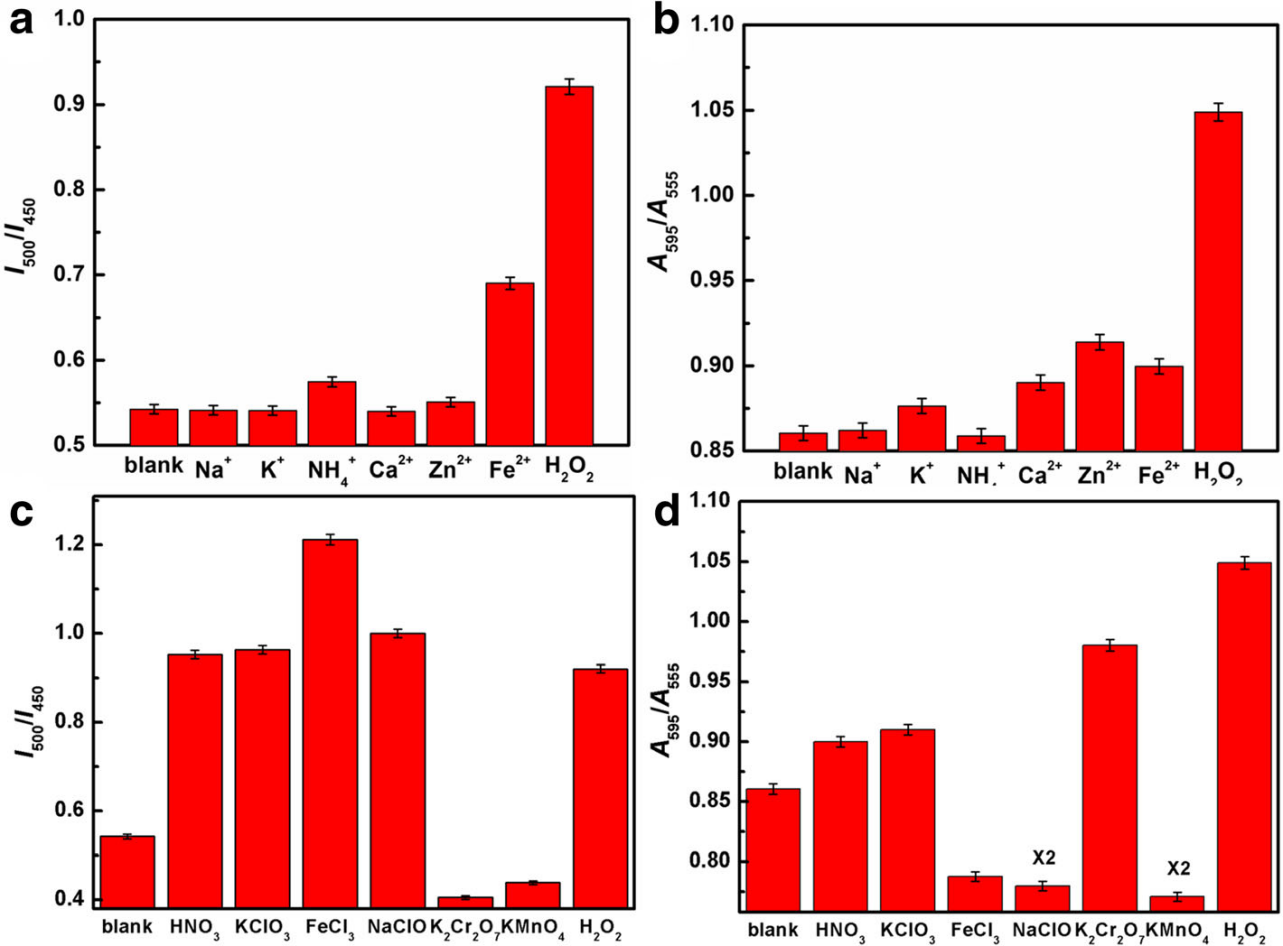
**a**, **c** Fluorescence ratio *I*_500_/*I*_450_ of solution containing CDs and various interferent cations (**a**) and oxidants (**c**). **b**, **d** Absorption ratio *A*_595_/*A*_555_ of solution containing CDs and various interferent cations (**b**) and oxidants (**d**)

To evaluate the selectivity of the nanosensors toward H_2_O_2_, interference assays were performed under identical conditions using some common cations, such as Na^+^, K^+^, NH^4+^, Ca^2+^, Zn^2+^, and Fe^2+^. As shown in Fig. 5a, b, the fluorometric and colorimetric changes of the CDs have been surveyed in the presence of different cations. In the presence of Na^+^, K^+^, NH_4_^+^, Ca^2+^, Zn^2+^, and Fe^2+^, the fluorescence ratio of *I*_500_ to *I*_450_ and the absorption ratio of *A*_595_ to *A*_555_ appear only as a slight variation compared with the blank sample, which mean these cations have little interference on the detection of H_2_O_2_. Moreover, we have also compared the impact on the CDs with other oxidants, such as HNO_3_, KClO_3_, FeCl_3_, NaClO, K_2_Cr_2_O_7_, and KMnO_4_ (Fig. 5c, d and Additional file 1: Figures S12 and S13), and we found that the fluorescence color changes from blue to green with the addition of these oxidants except K_2_Cr_2_O_7_ and KMnO_4_. So, we can rule out the interference from K_2_Cr_2_O_7_ and KMnO_4_ through the fluorescence change. In addition, we can easily exclude the impact from other oxidants like HNO_3_, KClO_3_, FeCl_3_, and NaClO from the result of the absorption ratio of *A*_595_ to *A*_555_. Hence, the dual-mode nanosensors demonstrated in this paper may be very promising in the high selectivity of the determination due to the synergistic effect of the two independent detection methods [30–33]. Furthermore, we have measured the response time of the fluorometric change upon the addition of H_2_O_2_ and found that the fluorescence decreases after injecting H_2_O_2_ and is kept stable at about 3.3 s (Additional file 1: Figure S14).

**Fig. 6:**
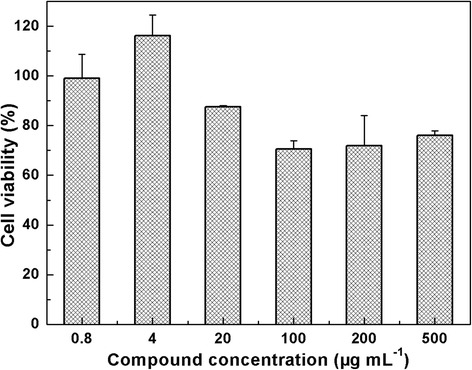
Cell viability of A549 cells after 48-h incubation in the different concentration of CDs

The viability of A549 cell was examined using standard CCK-8 assay for assessing the cytotoxicity of CDs. As shown in Fig. 6, we find that near 80% viability is obtained by incubating the A549 cells with CDs for 48 h even at high concentration of CDs like 500 μg mL^−1^. It is calculated that the 50% inhibitive concentration (IC50) of CDs is about 1106 μg mL^−1^ by the GraphPad Prism 5.0, which deduces the CDs have good biocompatibility and very low cytotoxicity at high concentration. Moreover, we have compared the analytical performance of previously reported nanosensors for the H_2_O_2_ determination shown in Additional file 1: Table S2. The biocompatibility, simplicity, and visualization of the detection are comparable to or even better than most of these reported H_2_O_2_ assays. Considering that the CD-based dual-mode nanosensors have a good selectivity toward H_2_O_2_ detection, the proper detection limit (LOD = 14 mM) at the same order with the blood glucose, and very low cytotoxicity at high concentration of CDs, the nanosensors are promising to be used in the test of blood glucose and other clinical requirements.

## Conclusions

In conclusion, we propose a dual-mode nanosensor based on CDs with both colorimetric and fluorometric output for the quantitative detection of H_2_O_2_ based on the fluorometric and colorimetric change of the CD solution upon the introduction of H_2_O_2_. The nanosensors are simple and facile to achieve naked-eye detection for H_2_O_2_. The mechanism of the nanosensors can be attributed to the fact that the external chemical stimuli like hydroxyl radicals from H_2_O_2_ bring about the change of surface properties and the aggregation of CDs, which dominate the emission and absorption of CDs. The proposed nanosensors exhibit good biocompatibility, high selectivity toward H_2_O_2_ with a linear detection range spanning from 0.05 to 0.5 M, and a detection limit of around 14 mM, which is comparable to the level of H_2_O_2_ produced by the GOD reactions. It is believed that the strategy reported in this paper may provide a promising approach for developing a novel sensor in blood glucose, which could be valuable in disease diagnosis and environmental testing.

## Additional file

Additional file 1:
**Fig. S1.** (a) TEM image of CDs after adding 0.5 M H_2_O_2_. (b,c) Histogram of distribution from TEM before (b) and (c) after adding 0.5 M H_2_O_2_. **Fig. S2**. Excited-emission matrix of the CDs with 2500 rpm (a), 5000 rpm(b), 7500 rpm(c) centrifugation and with the addition in 0.5 M H_2_O_2_ (d). **Fig. S3**. Absorption spectrum of the CDs normalized at 500 nm before and after adding 0.5 M H_2_O_2_. **Fig. S4**. XRD pattern of the CDs after adding 0.5 M H_2_O_2_. **Fig. S5**. Raman spectrum of the CDs after adding 0.5 M H_2_O_2_. **Fig. S6**. (a) Fluorescence stability of the CDs before and after adding 0.5 M H_2_O_2_ with the emission at 450 nm and 500 nm, respectively. (b) Photographic images of the CDs and the CDs under 1M H_2_O_2_ after 3 day. **Fig. S7**. FTIR spectra of the CDs before and after adding 0.5 M H_2_O_2_. **Fig. S8**. (a,b) XPS (full survey) of the CDs before (a) and after (b) adding 0.5 M H_2_O_2_. (c,d) XPS (C1s) of CDs before (c) and after (d) adding 0.5 M H_2_O_2_. (e,f) XPS (O1s) of CDs before (e) and after (f) adding 0.5 M H_2_O_2_. **Fig. S9**. The fluorescence intensity of the 112.5 μg mL-1 CDs diluted for 10, 20, 30 and 40 times. **Fig. S10**. The normalized fluorescence intensity of the CDs under different concentrations of H_2_O_2_ (0, 0.05, 0.1, 0.15, 0.25, 0.5, 1 and 2 M, from left to right). **Fig. S11**. The liner range of the absorption for sensing H_2_O_2_. **Fig. S12**. Fluorescence spectra of CDs in the presence of different oxidants. **Fig. S13**. UV-vis spectra of CDs in the presence of different oxidants. **Fig. S14**. The fluorescence intensity varied with time after injecting H_2_O_2_. **Tab. S1**. Limit of detection (LOD) of CDs sensor for H_2_O_2_. **Tab. S2**. Comparison of analytical performance of different nanosensors for H_2_O_2_ determination. (PDF 1384 kb)
