# Supplementary material for: Carbon Nanodots as Dual-Mode Nanosensors for Selective Detection of Hydrogen Peroxide
Source: Nanoscale Res Lett. 2017 Jul 6;12:447. doi: 10.1186/s11671-017-2214-6 (PMC5500605; doi:10.1186/s11671-017-2214-6)
Supplement: Additional file 1: — Fig. S1. (a) TEM image of CDs after adding 0.5 M H2O2. (b,c) Histogram of distribution from TEM before (b) and (c) after adding 0.5 M H2O2. Fig. S2. Excited-emission matrix of the CDs with 2500 rpm (a), 5000 rpm(b), 7500 rpm(c) centrifugation and with the addition in 0.5 M H2O2 (d). Fig. S3. Absorption spectrum of the CDs normalized at 500 nm before and after adding 0.5 M H2O2. Fig. S4. XRD pattern of the CDs after adding 0.5 M H2O2. Fig. S5. Raman spectrum of the CDs after adding 0.5 M H2O2. Fig. S6. (a) Fluorescence stability of the CDs before and after adding 0.5 M H2O2 with the emission at 450 nm and 500 nm, respectively. (b) Photographic images of the CDs and the CDs under 1M H2O2 after 3 day. Fig. S7. FTIR spectra of the CDs before and after adding 0.5 M H2O2. Fig. S8. (a,b) XPS (full survey) of the CDs before (a) and after (b) adding 0.5 M H2O2. (c,d) XPS (C1s) of CDs before (c) and after (d) adding 0.5 M H2O2. (e,f) XPS (O1s) of CDs before (e) and after (f) adding 0.5 M H2O2. Fig. S9. The fluorescence intensity of the 112.5 μg mL-1 CDs diluted for 10, 20, 30 and 40 times. Fig. S10. The normalized fluorescence intensity of the CDs under different concentrations of H2O2 (0, 0.05, 0.1, 0.15, 0.25, 0.5, 1 and 2 M, from left to right). Fig. S11. The liner range of the absorption for sensing H2O2. Fig. S12. Fluorescence spectra of CDs in the presence of different oxidants. Fig. S13. UV-vis spectra of CDs in the presence of different oxidants. Fig. S14. The fluorescence intensity varied with time after injecting H2O2. Tab. S1. Limit of detection (LOD) of CDs sensor for H2O2. Tab. S2. Comparison of analytical performance of different nanosensors for H2O2 determination. (PDF 1384 kb) [file 11671_2017_2214_MOESM1_ESM.pdf]

Supporting Information for:

Carbon nanodots as dual-mode nanosensors for selective detection of hydrogen peroxide

Cheng-Long Shen[1], Li-Xia Su[1], Jin-Hao Zang[1], Xin-Jian Li[1], Qing Lou[1*], Chong-Xin Shan[1,2*]

[1]*School of Physics and Engineering, Zhengzhou University, Zhengzhou 450052, China.*

[2]*State Key Laboratory of Luminescence and Applications, Changchun Institute of Optics, Fine Mechanics and Physics, Chinee Academy of Sciences, No. 3888 Dongnanhu Road, Changchun, 130033, China.*

List of Figures and Tables

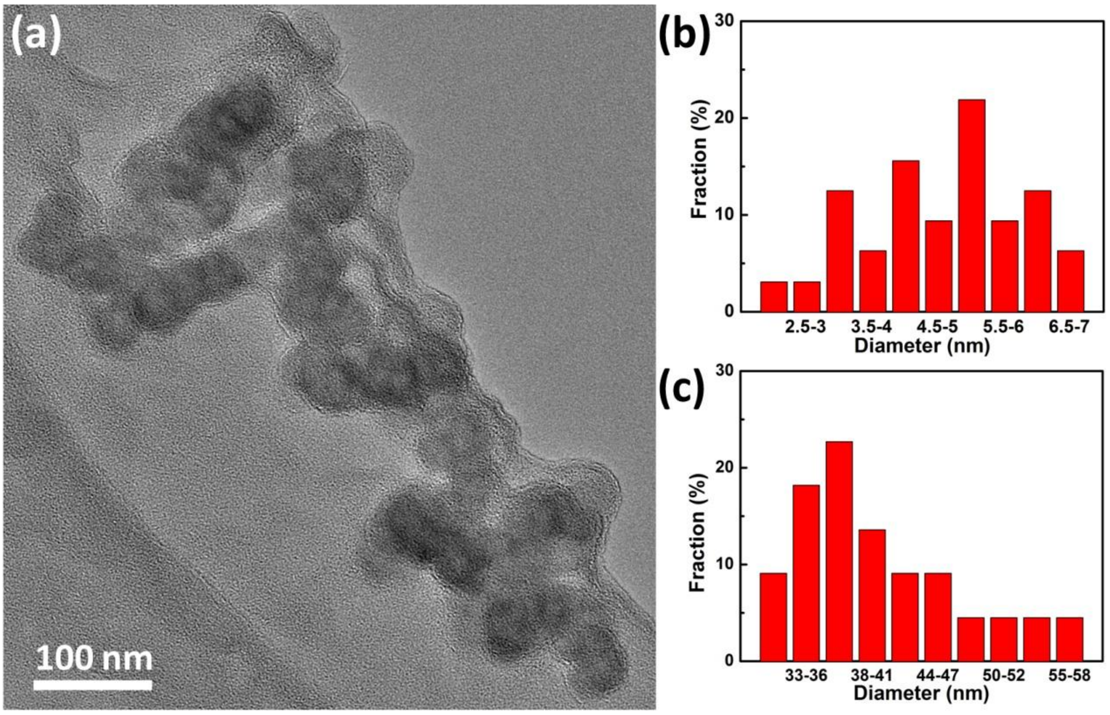

Figure S1. (a) TEM image of CDs after adding 0.5 M H_2O_2. (b,c) Histogram of distribution from TEM before (b) and (c) after adding 0.5 M H_2O_2.

From the histogram and TEM image, the diameters of the CDs after adding H_2O_2 enlarge about ten times, which are considered to be the aggregation of CDs from H_2O_2.

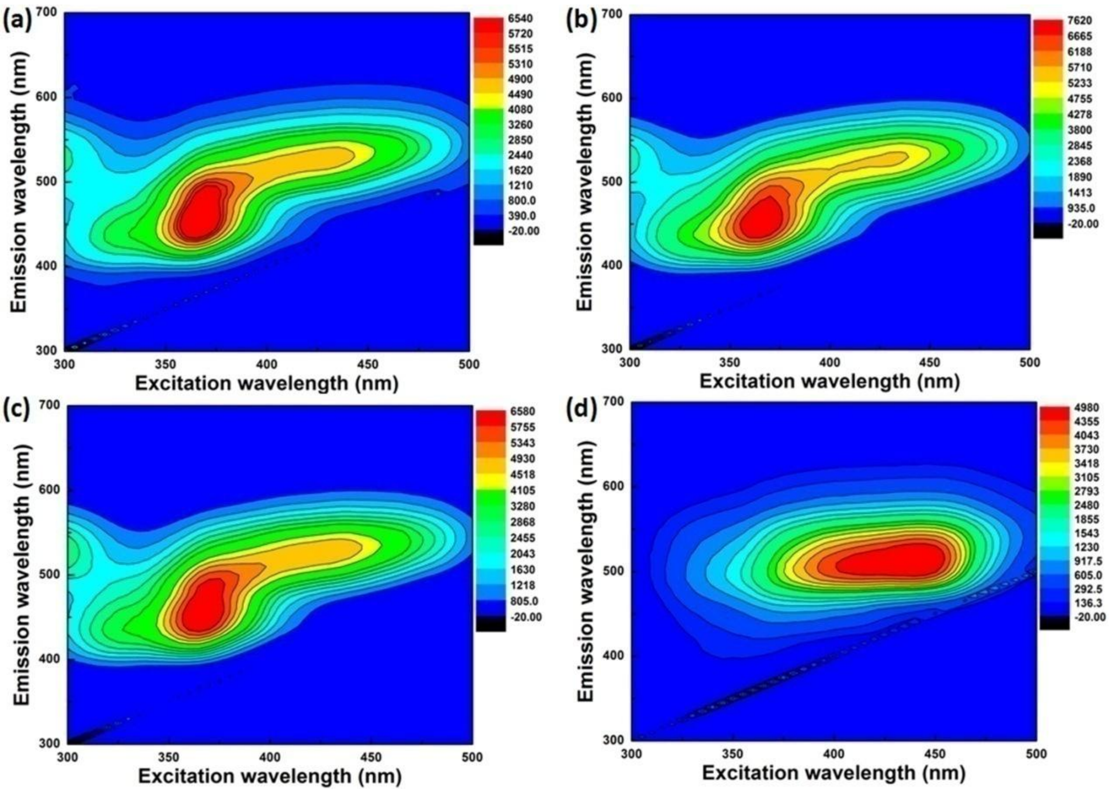

Figure S2. Excited-emission matrix of the CDs with 2500 rpm (a), 5000 rpm(b), 7500 rpm(c) centrifugation and with the addition in 0.5 M H_2O_2 (d).

The excited-emission matrix of the CDs with 2500 rpm, 5000 rpm and 7500 rpm centrifugation can prove the dual-emission fluorescence is from one kind of CDs with the same size.

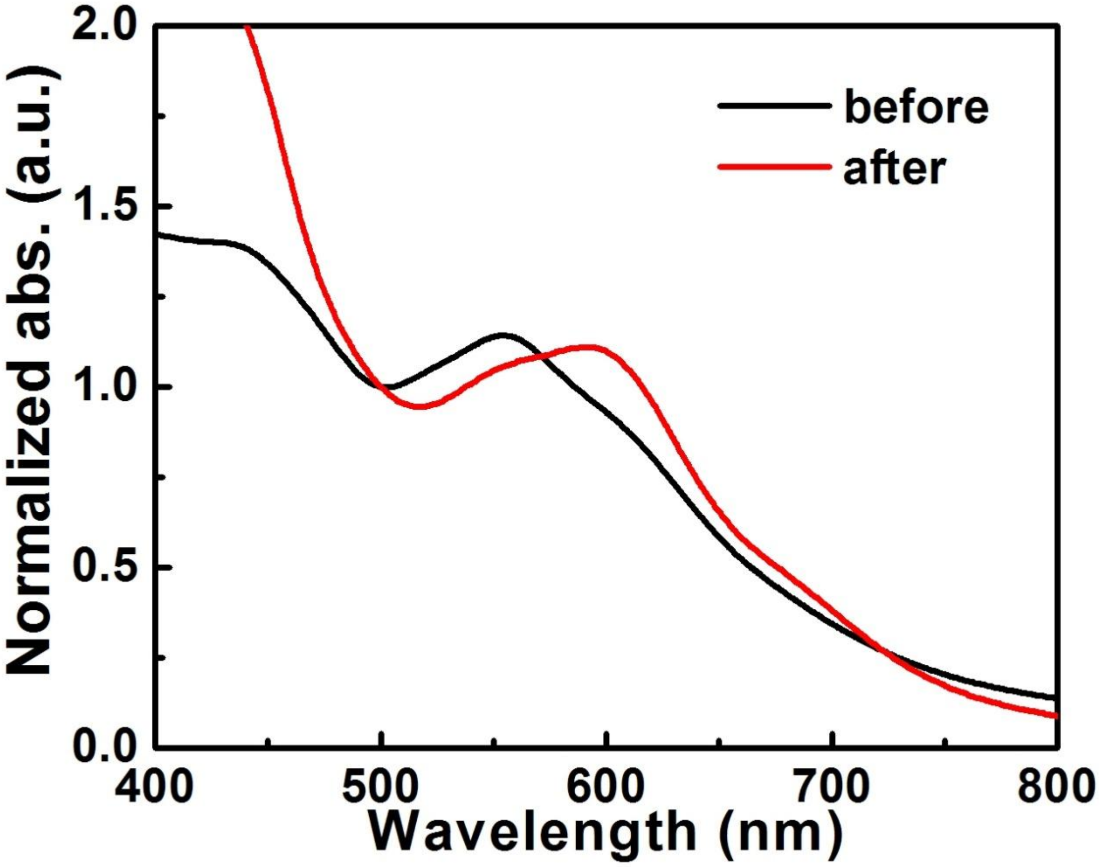

Figure S3. Absorption spectrum of the CDs normalized at 500 nm before and after adding 0.5 M H_2O_2.

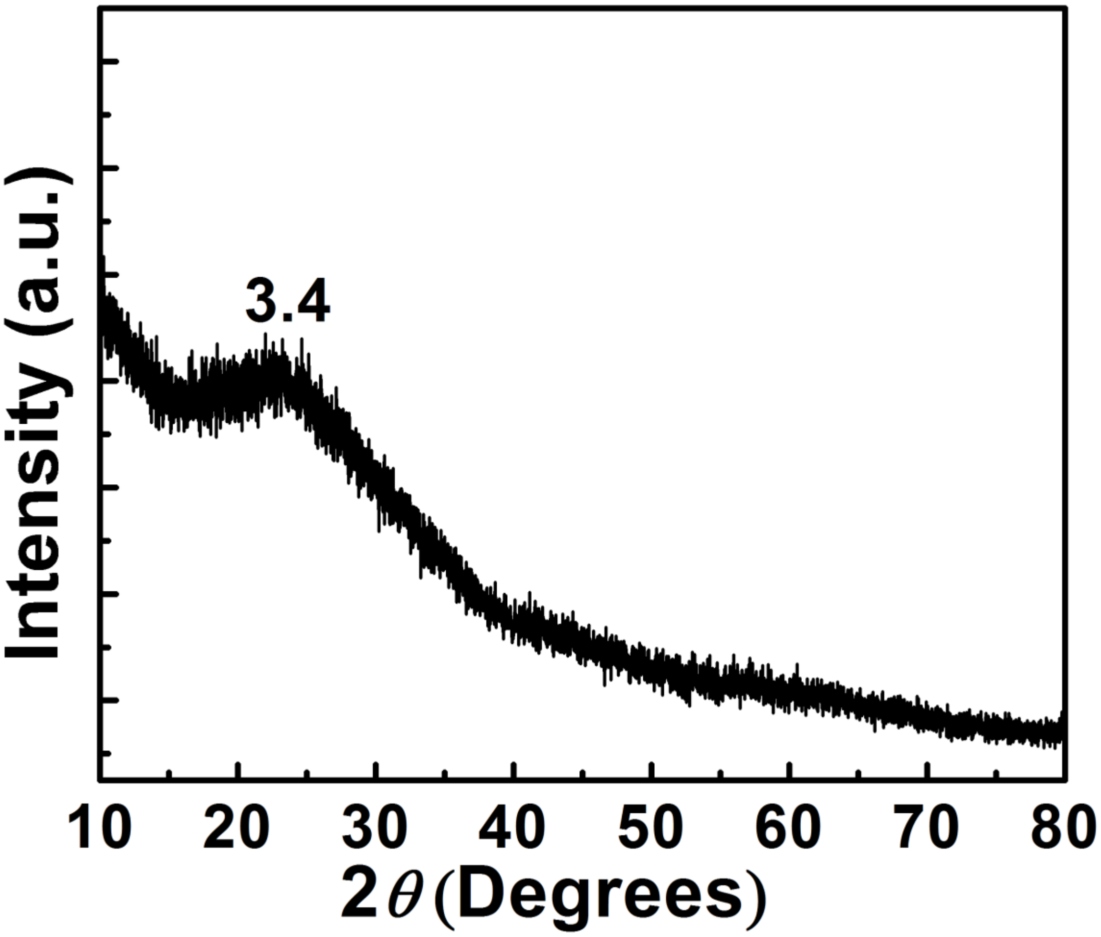

Figure S4. XRD pattern of the CDs after adding 0.5 M H_2O_2.

The XRD spectra of the CDs before and after adding H_2O_2 alter little.

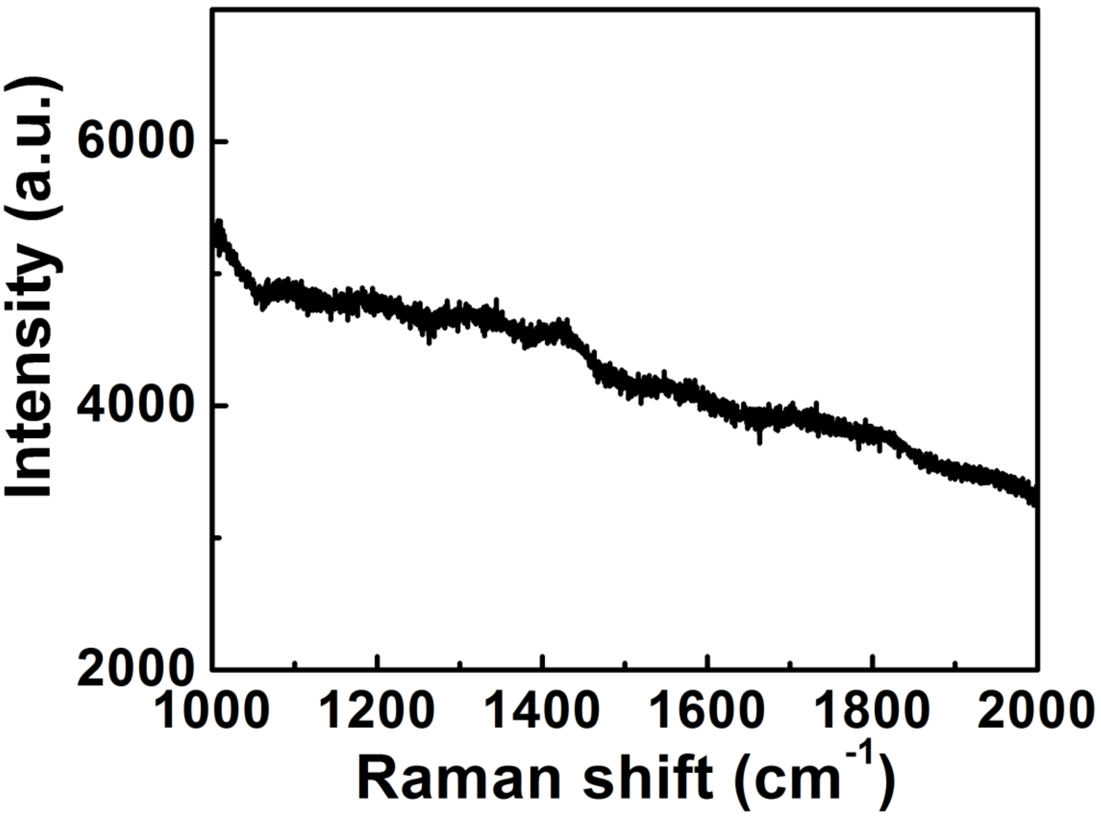

Figure S5. Raman spectrum of the CDs after adding 0.5 M H_2O_2.

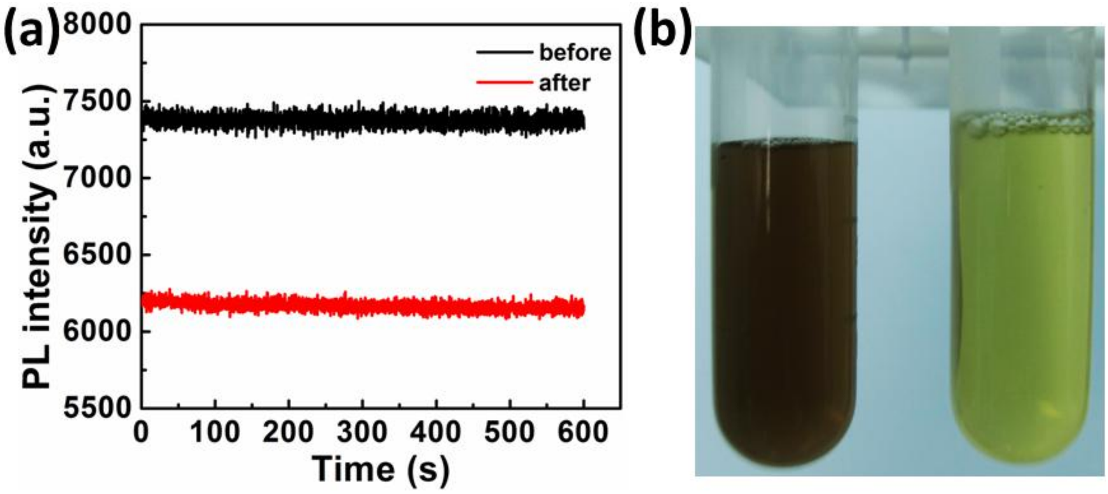

Figure S6. (a) Fluorescence stability of the CDs before and after adding 0.5 M H_2O_2 with the emission at 450 nm and 500 nm, respectively. (b) Photographic images of the CDs and the CDs under 1M H_2O_2 after 3 day.

The fluorescence stability of the CDs before and after adding H_2O_2 changes little.

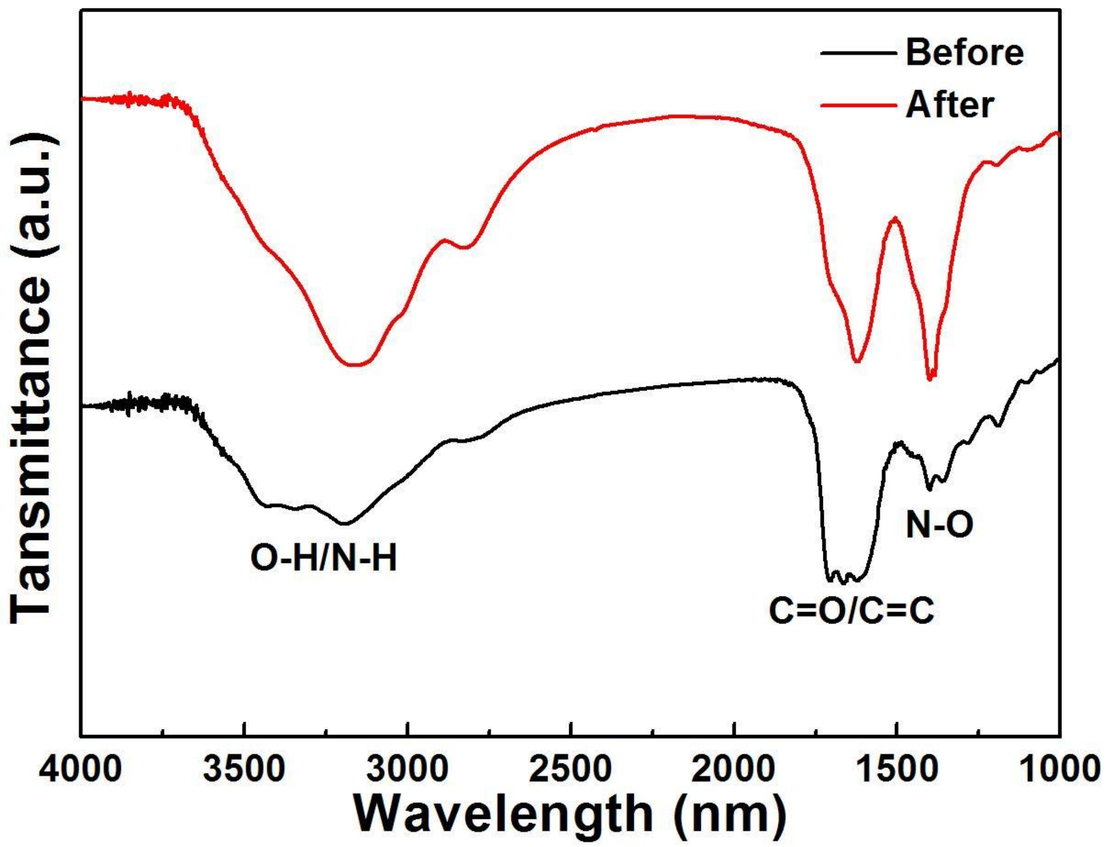

Figure S7. FTIR spectra of the CDs before and after adding 0.5 M H_2O_2.

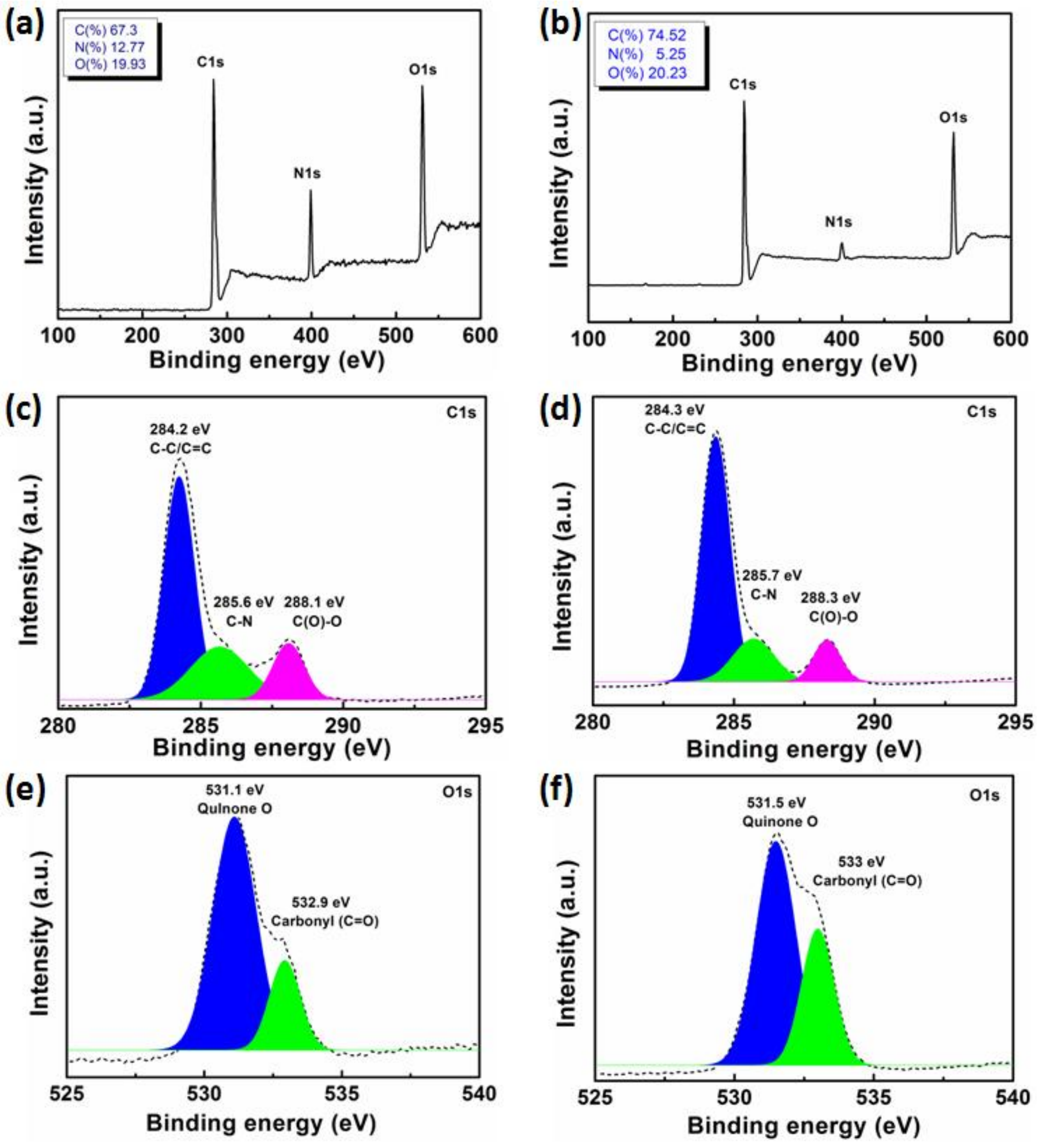

Figure S8. (a,b) XPS (full survey) of the CDs before (a) and after (b) adding 0.5 M H_2O_2. (c,d) XPS (C1s) of CDs before (c) and after (d) adding 0.5 M H_2O_2. (e,f) XPS (O1s) of CDs before (e) and after (f) adding 0.5 M H_2O_2.

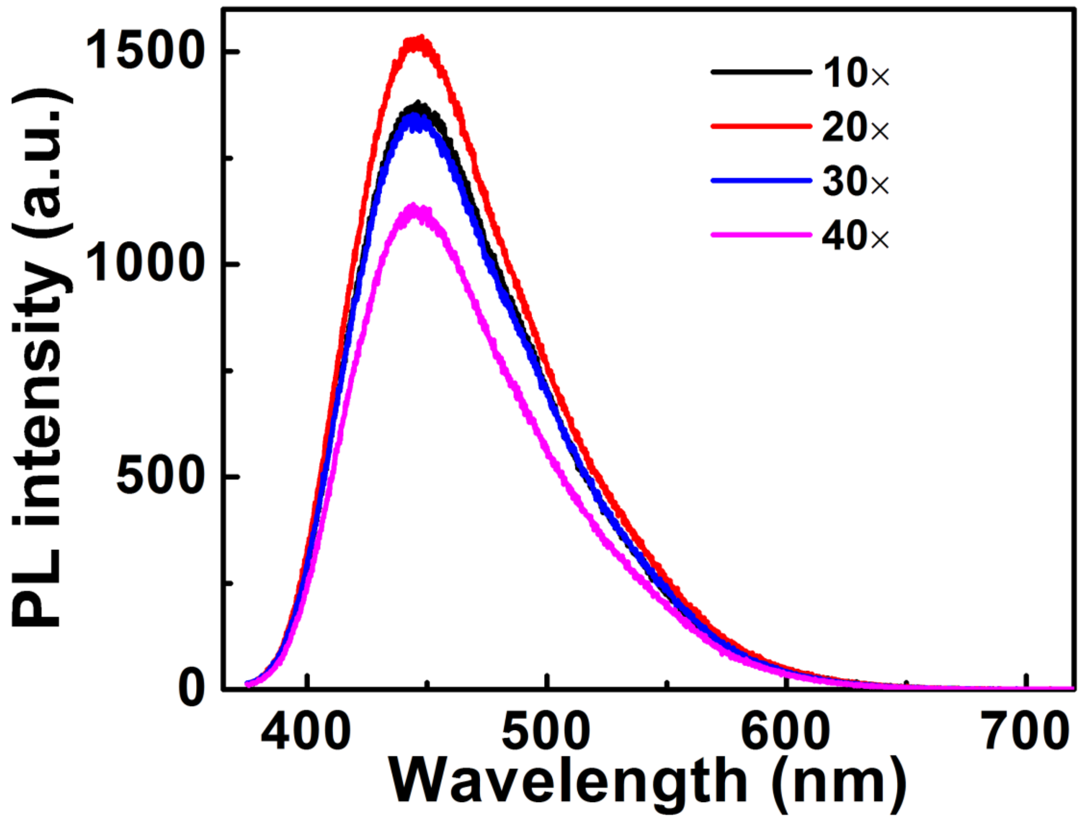

Figure S9. The fluorescence intensity of the 112.5 μg mL^{-1} CDs diluted for 10, 20, 30 and 40 times.

The high concentration may induce the fluorescence quenching of the CDs. So, the CDs should be diluted with a proper concentration (30x, 3.75 μg mL^{-1}) for increasing the accuracy of the nanosensor based on the CDs.

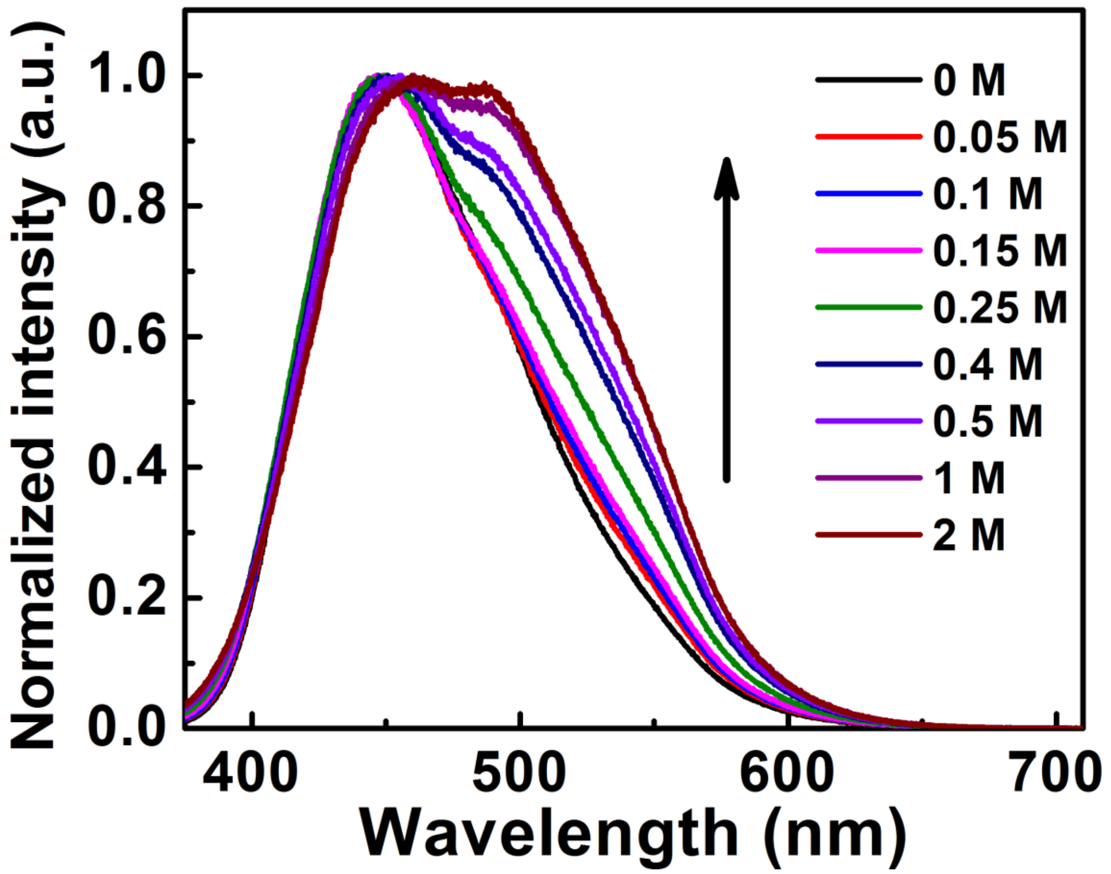

Figure S10. The normalized fluorescence intensity of the CDs under different concentrations of H_2O_2 (0, 0.05, 0.1, 0.15, 0.25, 0.5, 1 and 2 M, from left to right).

With increasing the concentrations of H_2O_2, the fluorescence intensity of the CDs at 500 nm obviously is enhanced.

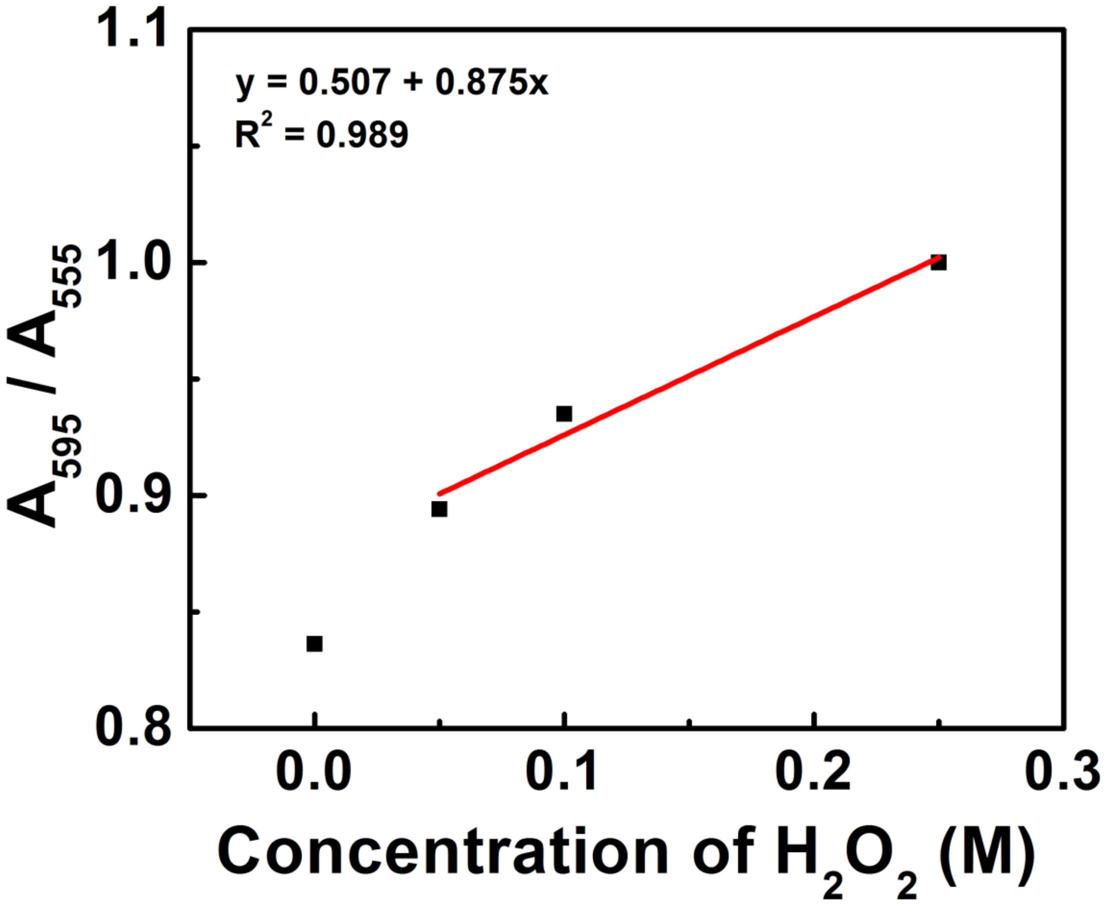

Figure S11. The liner range of the absorption for sensing H_2O_2.

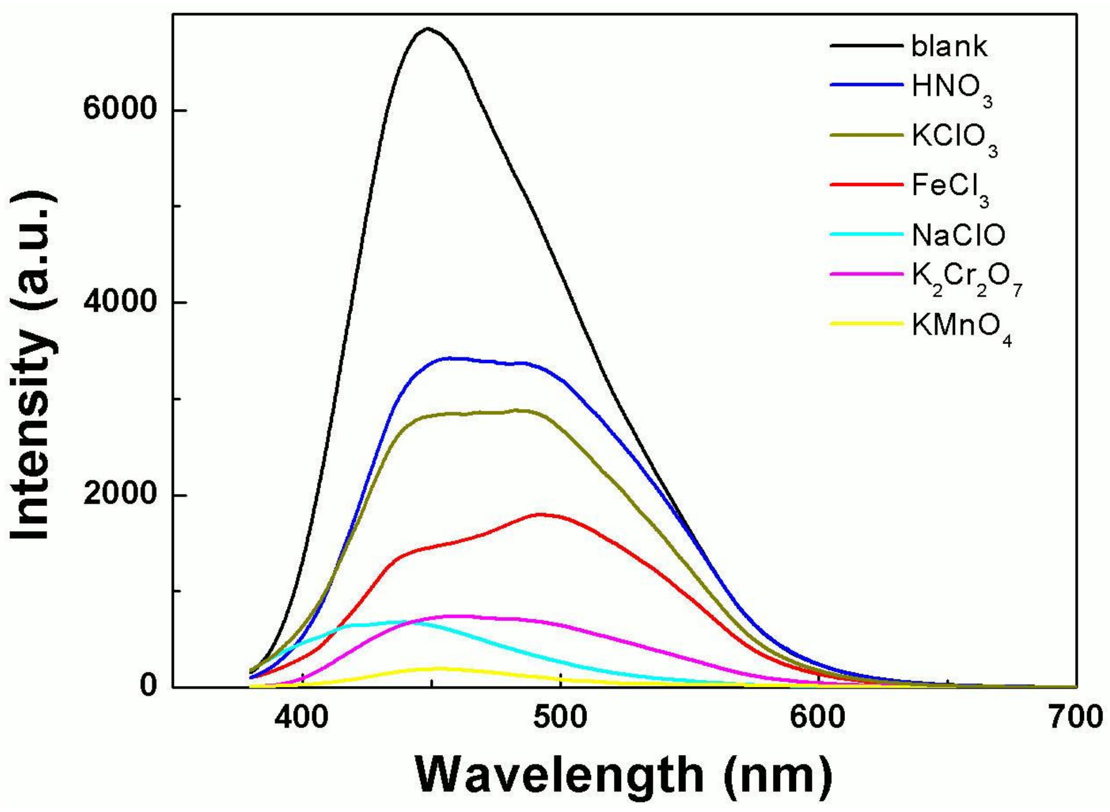

Figure S12. Fluorescence spectra of CDs in the presence of different oxidants.

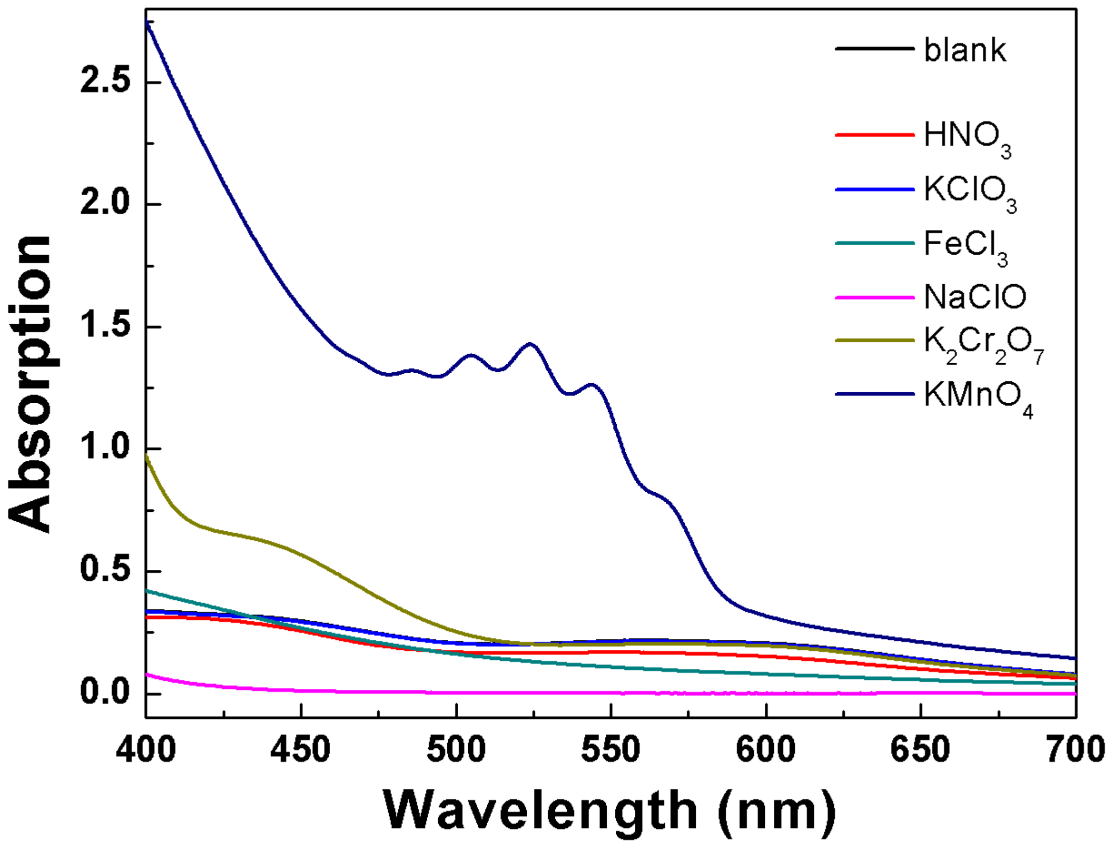

Figure S13. UV-vis spectra of CDs in the presence of different oxidants.

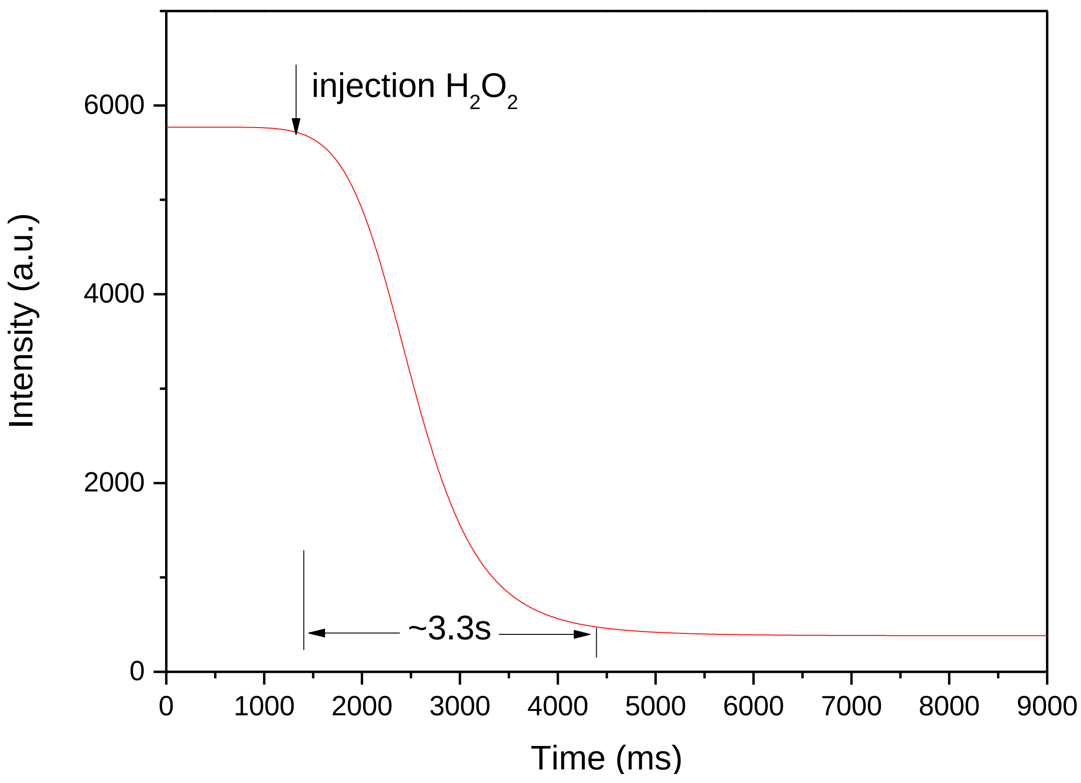

Figure S14. The fluorescence intensity varied with time after injecting H_2O_2.

Table S1. Limit of detection (LOD) of CDs sensor for H_2O_2

1	0.57062	11	0.57339
2	0.57068	12	0.57407
3	0.57217	13	0.5737
4	0.57029	14	0.57469
5	0.57011	15	0.57456
6	0.57134	16	0.5737
7	0.56819	17	0.573
8	0.57313	18	0.57362
9	0.57228	19	0.5729
10	0.56779	20	0.57937

The LOD rule of IUPAC: LOD = (k*b)/S

k=3, S was the slope of calibration curve, b was the tandard deviation of blank.

Calibration：S = 0.547，b = 0.00256，so LOD = 0.014M.

Table S2. Comparison of analytical performance of different nanosensors for H_2O_2 determination.

Sensing materials	Method	Linear range	LOD [a)]	Remark	Ref.
Au-Ag/C nanoclusters	Colorimetry	0.8-90, 90-500 μM	0.3 μM	Noble metals	1
SiO_2NPs-HRP [b)]	Colorimetry	1.2-72 μM	1.3 μM	Complicated operation with enzyme reaction	2
CNT/AgNPs	Electrochemistry	0.05-17mM	0.5 μM	Complicated operation and noble metals	3
Gr-CCS-AgNPs [c)]	Electrochemistry	20μM- 5.02mM	2.49μM	Complicated operation and noble metals	4
Au NC	Fluorescence	0.161-19.32 mM	20 μM	Difficulty for naked-eye detection and noble metals	5
dLys-AgNCs [d)]	Fluorescence	0.8-200μM	0.2μM	Difficulty for naked-eye detection and noble metals	7
polymer nanoparticles	Fluorescence	6-1000μM	2.0μM	Difficulty for naked-eye detection	9
CQD-HRP [e)]	Fluorescence	0.5-50 μM	0.2μM	Difficulty for naked-eye detection and operation with enzyme reaction	10
Carbon dots	Fluorescence and colorimetry	0.05-0.5M	50 mM	Simple and visualization by naked eye	This work

[a)] Limit of detection. [b)] SiO_2 nanoparticles and horse radish peroxidase. [c)] Silver nanoparticles selectively deposited on graphene-colloidal carbon sphere composite. [d)] Lysozyme-silver nanoclusters. [e)] Carbon quantum dots and horse radish peroxidase.

References:

1 L. Zhang, W. Hou, Q. Lu, M. Liu, C. Chen, Y. Zhang and S. Yao, *Anal. Chim. Acta*, 2016, **947**, 23-31.

2 J. Pla-Tolós, Y. Moliner-Martinez, C. Molins-Legua and P. Campins-Falcó, *Sensor. Actuat. B-Chem.*, 2016, **231**, 837-846.

3 W. Zhao, H.Wang, X. Qin, Z.Wang, Z. Miao, L. Chen, M. Shan, Y. Fang and Q. Chen, *Talanta*, 2009, **80**, 1029-1033.

4 H. Wang, H. Wang, T. Li, J. Ma, K. Li and X. Zuo, *Sensor. Actuat. B-Chem.*, 2017, **239**, 1205-1212.

5 P. Zhang, Y. Wang and Y. Yin, *Sensors*, 2016, **16**, 1124.

6 F. Liu, T. Bing, D. Shangguan, M. Zhao, and N. Shao, *Anal. Chem.*, 2016, **88**, 10631-10638.

7 H. Tan, C. Ma, Q. Li, L. Wang, F. Xu, S. Chen and Y. Song, *Analyst*, 2014, **6139**, 5516-5522.

8 Y. Zhang, X. Yang and Z. Gao, *RSC Adv.*, 2015, **5**, 21675-21680.
